# Skin-Inspired Pressure Sensor with MXene/P(VDF-TrFE-CFE) as Active Layer for Wearable Electronics

**DOI:** 10.3390/nano11030716

**Published:** 2021-03-12

**Authors:** Xiao-Quan Shen, Ming-Ding Li, Jun-Peng Ma, Qun-Dong Shen

**Affiliations:** Department of Polymer Science & Engineering and Key Laboratory of High Performance Polymer Materials & Technology of MOE, School of Chemistry & Chemical Engineering, Nanjing University, Nanjing 210023, China; xqshennju@163.com (X.-Q.S.); mingdingli2016@gmail.com (M.-D.L.); majunpeng001@gmail.com (J.-P.M.)

**Keywords:** electronic skin, capacitive pressure sensor, spinosum structure, MXene, high sensitivity, wearable electronics

## Abstract

Multi-functional electronic skin is of paramount significance for wearable electronics in health monitoring, medical analysis, and human-machine interfacing systems. In order to achieve the function of natural skin, mechanical sensing with high sensitivity is an important feature of electronic skin. Inspired by the spinosum structure under the skin, herein, we fabricate a new capacitive pressure sensor with two-dimensional transition-metal carbides and nitrides (MXene) and ferroelectric polymer (P(VDF-TrFE-CFE)) as an active layer and micropatterned Cr-Au deposited on polydimethylsiloxane as flexible electrodes. Such a method is facile, effective, easily operated, and low-cost. The device design provides great capacitive change as a consequence of large deformation under pressure. Benefiting from the randomly distributed microstructure and high dielectric constant of the active layer, the device demonstrates high sensitivity with great linearity (16.0 kPa^−1^ for less than 10 kPa), that is, a low detection limit of 8.9 Pa, and quick response. A series of dynamic physiological signals, including typing, knuckle motion, and voice recognition can be facilely detected, making it a competitive candidate in the field of wearable electronics.

## 1. Introduction

Electronic skins (E-skins) [1,2,3,4] have been attracting great attention in the past years due to their important roles in health monitoring [5,6], medical analysis [7], human-machine interfacing system [8], etc. Through continuous monitoring of external signals, it is able to feedback the real-time status of physiological and motion activities. Meanwhile, a large amount of information could be provided a straightaway platform to adjust our lifestyle by big data analysis. Pressure sensing is one of the most important features of E-skins and it is significant to convert the applied force into recognizable electronic signals to distinguish external stimulus. So far, mechanisms of pressure sensors are mainly divided into the following categories: piezoresistivity [9,10,11], capacitance [12,13], piezoelectricity [14,15], triboelectricity [16,17], and field effect-type [18]. Capacitive pressure sensors are considered one of the promising ways to monitor activities due to their well-established fabrication techniques, low power consumption, fast time response, and excellent temperature stability [19,20,21,22,23,24].

With the development of science and technology, a series of device structures, such as pyramids [25,26], pillars [27,28], hemispheres [29,30], and micro-porous [20], has been widely used to fabricate highly sensitive pressure sensors. These structures are realized by advanced microscale or nanoscale processing technologies, such as photolithography and 3D printing. Most recently, numerous efforts have been devoted to developing biomimetic pressure sensors with a multi-level structure. Human skin is such a sensor including epidermis, dermis, hypodermis, and related interfaces. For example, a spinosum-like graphene pressure sensor can reach a sensitivity of 25.1 kPa in a linearity of 0–2.6 kPa by abrasive paper template [31]. A flexible ferroelectric sensor based on a multilayer interlocked microdome geometry via a formwork casting process has an exceptionally broad pressure detection range (1.3 Pa–353 kPa) [11]. Jia et al. imitated human skin surface wrinkles and designed a new pressure sensor using reduced graphene oxide (rGO) films with continuous-gradient wrinkles [32]. The above skin-inspired strategies are often focused on piezoresistive sensors, which require continuous flowing electric current and thus high energy consumption [33]. Thus, it would be desirable to develop an effective and easily scalable structure to improve sensing performance in the capacitive sensors where almost no current flows.

In addition to the structural design of the capacitive sensors, the choice of the dielectric layers is very important as well. Conventional flexible polymer materials, such as polydimethylsiloxane (PDMS) [34,35], poly(methyl methacrylate) [36], and other elastomers [37,38], have been widely used for the dielectric layers in pressure sensing. These soft polymers exhibit large mechanical deformation but relatively low dielectric constant, that is, the ability to store the generated electric charge, which often restricts the further improvement of their sensitivity. Among thousands of polymers, ferroelectric polymers, including poly(vinylidene fluoride) (PVDF) and its binary or ternary copolymers, are more suitable for wearable electronics, since they have excellent electromechanical conversion performance and high capacity of electric storage [39]. Furthermore, adding a certain amount of fillers, such as barium titanate, graphene, carbon nanotubes, and metal particles into the polymer matrix to form nanocomposite is an effective strategy to improve the dielectric properties of the flexible polymer materials and preserve good flexibility at the same time [36,40,41,42,43].

MXene is a kind of two-dimensional (2D) transition-mental carbide, nitride, or carbonitride with the general formula of M_n+1_X_n_T_x_, where M represents an early transition metal, X represents carbon or nitrogen, n = 1, 2, or 3, and T_x_ is the surface functional groups (e.g., -OH, -O, and/or -F groups) [44,45]. Interestingly, MXene possesses an outstanding electronic conductivity, large surface area, and high aspect ratio, making it a great candidate to enhance the dielectric constant of the polymer [46,47,48]. More importantly, abundant polar groups on the surface of MXene greatly enhance its interactions with the polar polymer matrix. It leads to improved stability of the polymer composites free from tedious surface modification of the dielectric-reinforcing fillers in advance.

In this work, we demonstrate a flexible and wearable capacitive pressure sensor with polyethylene terephthalate (PET) as the substrate, MXene nanosheets/ferroelectric polymer nanocomposite as the dielectric layer, and micropatterned PDMS as the flexible electrodes, via a facile, effective, easily operated, and low-cost process. The MXene nanosheets in the ferroelectric polymer layer improve the capacity of electric storage, owing to their ultra-high conductivity and large aspect ratio. Meanwhile, in order to realize the high performance of pressure response, micropatterned PDMS deposited with Cr-Au serves as flexible electrodes by a secondary casting method. By applying pressure, the capacitive sensor exhibits high sensitivity with great linearity (16.0 kPa^−1^ for less than 10 kPa), a low detection limit of 8.9 Pa, and fast response. Furthermore, the device performs well in practical applications, such as non-contact sensing, knuckle motion, and voice recognition. It would be a competitive candidate in the field of wearable technologies and human-machine interfacing systems.

## 2. Experimental Section

### 2.1. Raw Materials

*N*, *N*-dimethylformamide (DMF, 99.5%), methylethyl-ketone (MEK, 99%), chloric acid (HCl, 36–38%), and lithium fluoride (LiF, 99.9%) were purchased from Sinopharm Co., Ltd. (Shanghai, China). Ti_3_AlC_2_ powder (400 mesh) was purchased from 11 Technology Co., Ltd. (Jilin, China). A terpolymer of vinylidene fluoride, trifluoroethylene, and chlorofluoroethylene, that is, P(VDF-TrFE-CFE), with a monomer ratio of 62.1:30.1:7.8 was purchased from Piezotech Arkema (France). Polyvinylidene fluoride (PVDF) was purchased from Bide Pharmatech Co., Ltd. (Shanghai, China). Polydimethylsiloxane (Sylgard 184) was obtained from Dow Corning Co., Ltd (Midland, MI, USA). Copper wire, gold (Au), chromium (Cr), and indium tin oxide /polyethylene terephthalate (ITO/PET) were purchased from Kaivo Optoelectronic Technology Co., Ltd (Zhuhai, China), with surface a resistance of 5.5–6.1 Ω sq^−1^. All chemicals were used as received unless otherwise noted.

### 2.2. Preparation of the Ti_3_C_2_T_x_ MXene Nanosheets

According to the previous reports, MXene nanosheets were synthesized by LiF/HCl selective etching process [44,45]. Briefly, 2 g of LiF and 40 mL of HCl solution (9 mol L^−1^) were stirred in a 100 mL of Teflon beaker for 15 min to form a homogeneous solution. Then, 2 g of MAX phase Ti_3_AlC_2_ powder was added slowly into the LiF/HCl aqueous solution, and the mixture was stirred for 24 h at 35 °C. After the Al layer was etched, the reaction products were repeatedly washed and centrifuged in deionized water (3500 rpm, 10 min), until the supernatant pH was greater than 6. The supernatant was removed, and ethanol was added to the precipitate by ultrasound at room temperature for 1 h, and the precipitate was collected by centrifugation (10,000 rpm, 10 min). The above product was redispersed in the deionized water and sonicated for 20 min in an ice bath. Finally, the dark green supernatant was collected as MXene nanosheets by centrifugation (3500 rpm, 5 min).

### 2.3. Fabrication of the Dielectric Layer

The prepared MXene nanosheets and P(VDF-TrFE-CFE) powder (5/95 wt/wt) were dispersed in DMF and MEK (mass ratio of 1:1) with a content of 100 mg mL^−1^ and stirred at room temperature for 24 h. Then the dielectric layer was fabricated in a nitrogen atmosphere by spin-coating the dispersion on the ITO/PET film at 800 rpm for 10 s and 3000 rpm for another 60 s. After spin-coating, the dielectric layer was annealed in a vacuum at 120 °C for 2 h to remove the residual solvents.

### 2.4. Fabrication of the Micropatterned Electrode

An abrasive paper used as a template was cut into a size of 1.5 × 1.5 cm^2^. In order to reduce the adhesion, the template was immersed in the formula solution (10 wt% detergent/75% ethanol) for 30 min and dried for another 1 h to completely remove the moisture. PDMS with a base to crosslinking agent mass ratio of 5:1 was prepared and placed at room temperature until bubbles totally disappeared. Then an appropriate amount of PDMS mixture was poured on the template with a surface roughness of No. 180. After curing at 70 °C for 1 h, the PDMS reverse mold was stripped off and re-cured at 120 °C for another 1 h to form a second molding template. The second molding process was similar to the first molding process, except that the weight ratio of base to crosslinking agent was 10:1, and the curing condition was at 80 °C for 1.5 h. After two molding processes, the PDMS film with the pattern of the abrasive paper was fabricated. Then, 5 nm chromium and 40 nm gold were deposited by means of thermal evaporation on the micropatterned PDMS film.

### 2.5. Assembly of the Capacitive Sensor

The dielectric layer was put on the micropatterned PDMS electrode. Then, two copper wires were connected to the top and bottom electrodes through a silver paste. A MXene/(PVDF-TrFE-CFE) capacitive pressure sensor was thus assembly through the simple steps above.

### 2.6. Characterization

The crystal structure and properties of the Ti_3_C_2_T_x_ MXene nanosheets were investigated with an X-ray powder diffractometer (XRD, Bruker D8 ADVANCE, Berlin, Germany), transmission electron microscope (TEM, JEOL JEM-2100, Akishima, Japan), and dynamic light scattering (DLS) laser particle sizer (Brookhaven 90Plus, Holtsville, NY, USA). Raman spectroscopy was conducted on a Horiba Aramis Raman microscope with 785-nm laser excitation. X-ray photoelectron spectroscopy (XPS) was collected by Japan ULVAC PHI5000 Versa Probe. The morphology of the MXene nanosheets and MXene/P(VDF-TrFE-CFE) sensor was characterized by a field-emission scanning electron microscope (SEM, Japan Hitachi S-4800). Dielectric properties of the dielectric layer were obtained by a broadband dielectric spectrometer (Germany Novocontrol Concept 80). A testing machine (SANS, CMT4204, Shenzhen, China) was used to apply pressure to the sensor and record the values. The capacitance change was measured by an inductance capacitance and resistance (LCR) meter (TONGHUI, TH2830, Changzhou, China) with a bias of 250 mV.

## 3. Results and Discussion

### 3.1. Design and Fabrication of the Bioinspired Capacitive Sensor

**Figure 1:**
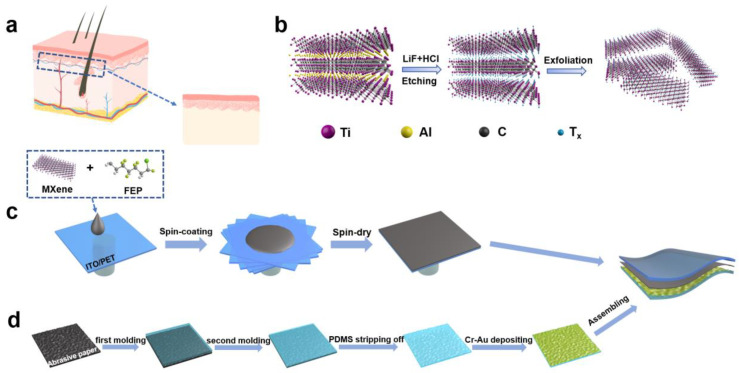
(**a**) The cross-section of human skin and enlarged spinosum structure between the dermis and the epidermis. (**b**) The etching process of MXenes from the Ti_3_AlC_2_ powder (MAX phase). (**c**,**d**) Schematic illustrating the fabrication steps of MXene/P(VDF-TrFE-CFE) capacitive pressure sensor.

The skin as one of the largest organs covers the surface of our body for acquiring external stimulation, as shown in Figure 1a. Merkel’s discs in the upper surface of the skin are the receptors for gentle touch, and subsequently transmit the tactile signals to the nerve, leading to a physiological electric signal transmittable to our brain. Such natural electromechanical sensors in large numbers have a spinous microstructure responsible for the high sensitivity to touch signals, due to local stress enhancement. An abrasive paper has a special geometry similar to that of the spinosum under the skin, thus it can be used as a facile template to reproduce the sensitivity-enhancing microstructure.

In addition to structural factors, a flexible material with a large electromechanical response is also necessary for biomimetic skin. Ferroelectric polymers, for example, PVDF and P(VDF-TrFE-CFE), are one of the most promising materials for high-performance electromechanical transduction [49] and electric energy storage devices [50]. Typically, the dielectric capacitance change of P(VDF-TrFE-CFE) in response to tactile stimuli is relatively small, thus a field-effect device is inevitable to read out the generated electric signals. A universal way to enhance dielectric properties is adding semiconducting or conducting fillers into the polymer matrix to form nanocomposites [51,52,53]. MXene, a famous two-dimensional material, known for its excellent electronic properties and nanostructure, is thus suitable for improving capacitance and consequent sensitivity of the biomimetic skin. As shown in Figure 1b, Ti_3_C_2_T_x_ MXene nanosheets are prepared in a mixed solution of LiF/HCl at mild temperature by etching the Al layer of MAX phase Ti_3_AlC_2_ and intercalating of specific solvent. Then, the prepared Ti_3_C_2_T_x_ MXene nanosheets and P(VDF-TrFE-CFE) powder are blended in DMF and MEK to form a uniform dispersion. The fabrication of a bioinspired capacitive sensor mainly contains three steps (Figure 1c,d): fabrication of the dielectric composite layer, fabrication of micropatterned electrode, and assembly of the device. In the first step, according to different sensing requirements, the corresponding size of an ITO/PET substrate is selected. In this work, considering the fit with the human body, a 1.5 cm × 1.5 cm substrate was cut. Then, MXene/P(VDF-TrFE-CFE) mixed dispersion is dropped on the substrate and spin-coated into the film. In the second step, inspired by the micro-spine structure of human skin, an abrasive paper is used as a template to fabricate micropatterned PDMS. When the cast film is peeled from the mold, anti-sticking treatment is very necessary. Dip-coating the mold with a formula solution (10 wt% detergent/ 75% ethanol) can form a molecular layer to reduce the interaction between the materials. After two molding processes, 5 nm Cr and 40 nm Au are deposited on the PDMS film with the pattern of the abrasive paper. Other materials, such as PEDOT/PSS, rGO, and Ag NWs, can also be used as electrodes. In the final step, the dielectric layer is put on a PDMS micropatterned electrode to constitute the sensor (Figure S1). In order to facilitate the reading of electrical signals, the top and bottom electrodes are connected with two copper wires through a silver paste.

### 3.2. Structure of MXenes and the Bioinspired Capacitive Sensor

**Figure 2:**
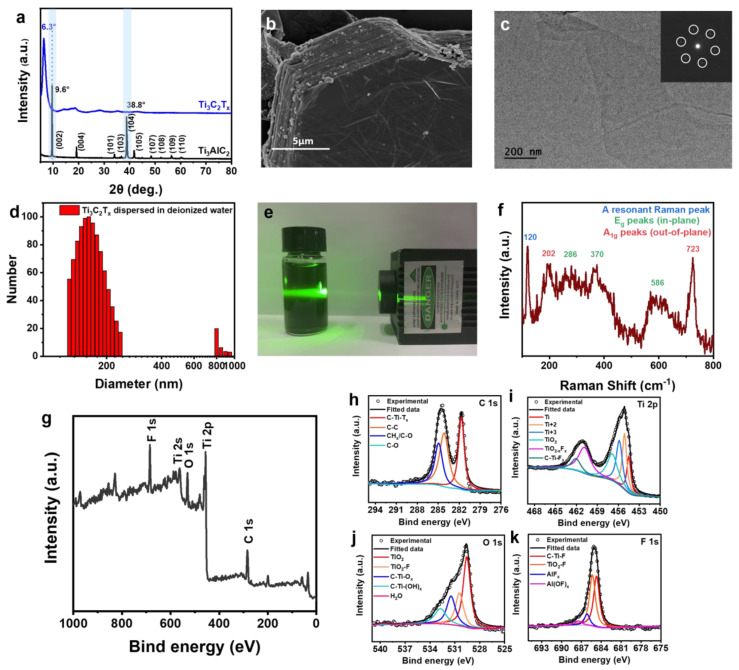
(**a**) XRD patterns of Ti_3_C_2_T_x_ MXene nanosheets and Ti_3_AlC_2_ powder. (**b**) SEM image of MXene after etching (**c**) TEM image of MXene nanosheet. Inset: Corresponding SAED pattern. (**d**) The size distribution of Ti_3_C_2_T_x_ MXene nanosheets. (**e**) The Tyndall effect of MXene colloidal solution in water. (**f**) Raman spectrum of Ti_3_C_2_T_x_ MXene film (785 nm laser). (**g**–**k**) X-ray photoelectron spectroscopy (XPS) of (**g**) Ti_3_C_2_T_x_ MXene and corresponding high-resolution spectra of (**h**) C 1s, (**i**) Ti 2p, (**j**) O 1s, and (**k**) F 1s.

MXene nanosheets are synthesized from MAX phase Ti_3_AlC_2_ with LiF and HCl and exfoliated by ultrasonication. A typical XRD pattern is shown in Figure 2a. The disappearance of the (104) peak of Ti_3_AlC_2_, which is located at 38.8°, reveals the successful etching of the Al atomic layer from Ti_3_AlC_2_ and preparation of Ti_3_C_2_T_x_ MXene nanosheets. The (002) strong peak shifts from 9.8° to 6.3°, which suggests the highly uniform lamellar structure and extended interlayer spatial distance (Ti_3_AlC_2_: 0.92 nm, Ti_3_C_2_T_x_: 1.40 nm). After etching and before exfoliation, the morphology of MXene has a significant change from a block (Figure S2) into an accordion-like layered crystal structure (Figure 2b). The delaminated MXene nanosheets are fabricated after exfoliating with the help of ethanol by ultrasonication. As shown in Figure 2c, the TEM image and corresponding selected-area electron diffraction (SAED) pattern indicate the hexagonal crystal and paper-like structure of the MXene nanosheets. The MXene nanosheets have an average size of 165 nm according to dynamic light scattering (DLS) measurement (Figure 2d). Meanwhile, the MXene nanosheets are stably dispersed in the deionized water to afford a colloidal solution that has a Tyndall effect (Figure 2e). A resonant Raman peak and corresponding Raman-active modes (E_g_ and A_1g_) of the MXene also can be found in the Raman spectrum and confirm the successful preparation of MXene (Figure 2f). In order to understand the surface elemental composition of the MXene nanosheets, XPS was conducted to identify the presence of C 1s, Ti 2p, O 1s, Ti 2s, and F 1s peaks, which are located at 284, 456, 530, 563, and 685 eV, respectively (Figure 2g–k). Therefore, there is a large amount of hydroxide (-OH, -O-) and fluoride groups (-F) on the surface of the MXene nanosheets, which is beneficial for the formation of abundant hydrogen bonds and van der Waals interactions with fluoride groups on P(VDF-TrFE-CFE).

**Figure 3:**
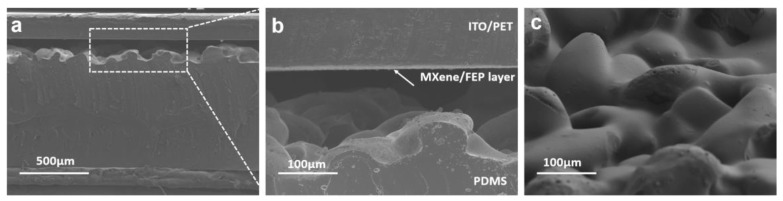
(**a**) Cross-sectional SEM image of MXene/P(VDF-TrFE-CFE) capacitive sensor. (**b**) Enlarged SEM image corresponding to the indicated region in (**a**). (**c**) Oblique top view of micropatterned PDMS micropatterned electrode.

Figure 3a shows the cross-section structure of the bioinspired capacitive sensor, and the nether is micropatterned PDMS while the upper is dielectric layer @ITO/PET substrate. The average thickness of the micropatterned PDMS is approximately 818 μm. Enlarged SEM images of the interface between the nether and the upper are shown in Figure 3b, it is obvious to notice that there is a thin dielectric layer on ITO/PET substrate. The surface morphology of PDMS appears as a disorderly protrusion, which is similar to the spinosum structure under human skin, as shown in Figure 3c. Furthermore, a three-dimensional (3D) optical profiler (Figure S3a,b, Table S1) reveals that PDMS with micropatterned electrodes has a nonuniform height distribution within the range of 0–119 μm. Thus, the contact between the dielectric layer and PDMS micropatterned electrode can be changed by venting or filling air, which leads to the change of capacitance during applying or removing the external force, providing the basis for the capacitive sensors.

### 3.3. Sensing Performance of the Bioinspired Capacitive Sensor

**Figure 4:**
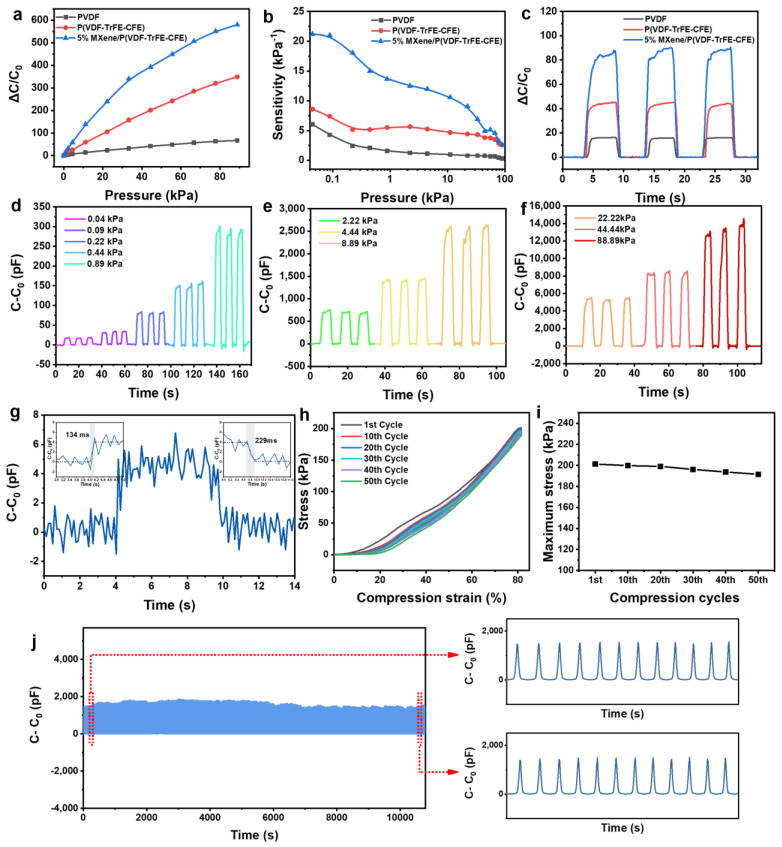
Basic sensing evaluations of MXene/P(VDF-TrFE-CFE) capacitive pressure sensor. (**a**,**b**) Relative change in (**a**) capacitance and (**b**) sensitivity versus pressure of the capacitive pressure sensor based on MXene/P(VDF-TrFE-CFE), P(VDF-TrFE-CFE), and PVDF dielectric layer. (**c**) The response of capacitance-time curves under the pressure of 8.89 kPa. (**d**–**f**) The ΔC-T curves of the sensor using MXene/P(VDF-TrFE-CFE) dielectric layer under serial pressures ranging from 0.04 to 88.89 kPa. (**g**) Relative capacitance variation of the pressure sensor under micro-pressure of 8.9 Pa. Inset: the corresponding response and recovery time. (**h**) Stress-compression strain curve and (**i**) maximum stress of the sensor for different cycles. (**j**) Dynamic press-release cyclic test under 5.0 kPa and magnified capacitance signals at the beginning and the ending, respectively.

In order to evaluate the performance of the sensor, three different dielectric layers are used for comparison, as shown in Figure 4. Generally, the pressure sensitivity (S) is an important parameter of the devices, which can be defined as S = (ΔC/C_0_)/ΔP, where C is the capacitance of the sensor and P is the applied pressure. As shown in Figure 4a, it can be seen intuitively from the ΔC/C_0_-ΔP curve that the bioinspired sensor based on three different dielectric layers exhibits excellent pressure-sensing performance. The sensor using MXene/P(VDF-TrFE-CFE) has the most obvious signal change than the other two when the external pressure increases. Figure 4b shows the sensitivity curve at each loading stage. At the initial stage, where the applied pressure is small, the difference in sensitivity is largest (21.2, 8.6, and 6.0 kPa^−1^ for MXene/P(VDF-TrFE-CFE), P(VDF-TrFE-CFE), and PVDF, respectively), which is ascribed to the difference in the effective dielectric capacitance. For the capacitive sensor based on MXene/P(VDF-TrFE-CFE), the average sensitivity below 10 kPa is 16.0 kPa^−1^, which is much better than the others (6.6 kPa^−1^ and 3.5 kPa^−1^ for P(VDF-TrFE-CFE) and PVDF, respectively). This reveals that the MXene-doped dielectric layer is beneficial to the increase in sensitivity. The sensitivity becomes smaller under higher pressure, especially beyond 40 kPa, and it may be attributed to the saturation of distance between two electrodes, which is determined by the elastic modulus, Poisson’s ratio, and density of materials. In practical applications, high sensitivity is not required for higher pressure, and it is desirable to increase the detectable range as the sensitivity decreases.

Figure 4c shows the real-time behaviors of the devices under a constant pressure of 8.89 kPa for a few seconds, the relative capacitance change of MXene/P(VDF-TrFE-CFE) is nearly twice that of P(VDF-TrFE-CFE). It is worth noting that, immediately after the application of force, the curve rises rapidly at first, then rises slowly. The reason for this phenomenon is the existence of the polymer, especially PDMS. At the moment of applying pressure, the chain in the polymer undergoes a conformational transformation, causing a general elastic deformation; and then the segments or molecular chains slip, slowly reaching a steady state.

Since high sensitivity is beneficial for the pressure resolution capability of the sensor, in Figure 4d–f, a series of different pressures with gradients is applied to the capacitive pressure sensor. As the pressure increases, the capacitance gradually increases, indicating that the air in the sensor is gradually exhausted and the total capacitance increases. Therefore, the sensor can easily distinguish the strength of the applied pressure by judging the value of the capacitance. At the same time, the detection limit and the response time are two other important parameters of the pressure sensor. Figure 4g shows that the sensor can detect light objects with pressure as low as 8.9 Pa. In addition, the corresponding response and recovery time of the capacitive pressure sensor are 134 ms and 229 ms, respectively. The elastic deformation of the PDMS is an important factor leading to the hysteresis of time, but this does not hinder the application in practice. The durability and stability of the capacitive sensor are also important, as shown in Figure 4h, the hysteresis test of the sensor is conducted, and the stress-compression strain curve proves good mechanical behaviors of the sensor. It can be seen that the maximum stress of the sensor still retains ~80%, which is from 201.29 kPa (1st cycle) to 191.57 kPa (50th cycle) (Figure 4i). Furthermore, Figure 4j shows ~1500 cycles of a loading-unloading test under 5.0 kPa, and the fluctuations of the capacitance output are rather small. Meanwhile, it can be seen from the magnified insets that there is no significant difference in the amplitude of the initial and the final, which strongly confirms the long-period usage of the sensor.

### 3.4. Sensing Mechanism of the Bioinspired Sensor

**Figure 5:**
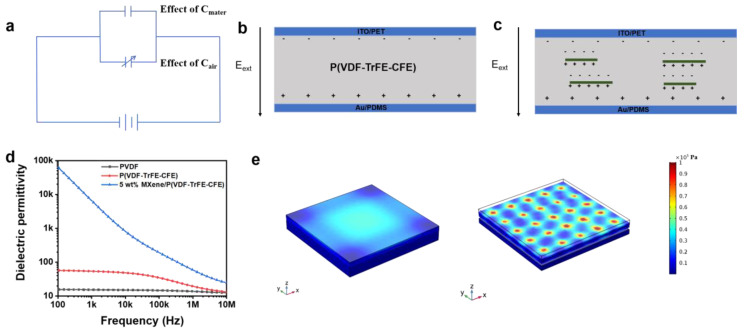
(**a**) Equivalent circuit diagram of the capacitive pressure sensor based on MXene/P(VDF-TrFE-CFE). (**b**,**c**) Schematic illustration of the polarization charges distribution in P(VDF-TrFE-CFE) and MXene/P(VDF-TrFE-CFE) film, respectively. (**d**) Dielectric permittivity of PVDF, P(VDF-TrFE-CFE), and MXene/P(VDF-TrFE-CFE) films. (**e**) Comsol multi-physics field simulation showing stress distribution and relative deformation of PDMS with non-pattern and pattern at an external load pressure of 5 kPa.

In general, the capacitance of a capacitor is related to the permittivity (ε) of the dielectric layer, the effective area of two electrodes (A), and the distance between the plate electrodes (d), which is expressed as C = Aε/d. In order to illustrate the mechanism of the capacitive pressure sensor based on MXene/P(VDF-TrFE-CFE) more vividly, Figure 5a shows the equivalent circuit diagram of the capacitive sensor. The permittivity of the materials and air between electrodes represents the inherent capacitance. In the system of sensor, the expression of the capacitance can be further written as $C=C_{mater}+C_{air}=f_{mater}A\varepsilon_{mater}/t_{mater}+f_{air}A\varepsilon_{air}/t_{air}$, where *f* represents the volumetric fraction of each component and *t* is the thickness of each layer. Before applying pressure on the sensor, there is a large amount of air between the parallel electrodes. At the time, due to the large distance between the parallel electrodes, the capacitance of the whole system is mainly determined by air, behaving quite small. In the presence of external pressure, the air is removed, and the capacitance is increased by the increasing volumetric fraction of the MXene/P(VDF-TrFE-CFE) layer. The microscopic model can effectively describe the increased sensitivity of the sensor based on the MXene/P(VDF-TrFE-CFE) layer. As shown in Figure 5b,c, in this model, the polarization charge is accumulated near the parallel electrodes of the P(VDF-TrFE-CFE) layer when an electric field is applied. By contrast, it can be seen that charges accumulate on the interfaces between the MXene sheets and the polymer matrix. Hence, the charge capacity of the dielectric layer increases, leading to a larger change in capacitance of the sensor. The capacitance change will be more obvious as the dielectric permittivity increases, which is the basis for improving the sensitivity. Here, P(VDF-TrFE-CFE) is chosen as the dielectric layer, because it has large intrinsic permittivity (54.5), which is one of the highest among known polymers, as well as flexibility. By doping MXene into the P(VDF-TrFE-CFE) matrix, the permittivity increased sharply to 6718 with a mass content of 5% (Figure 5d). Another important factor is the distance between the plate electrodes. To achieve greater displacement, the micropatterned structure is introduced into the device because the presence of more air volume at the interface will lead to greater deformation. Finite element modeling (FEM) is conducted by COMSOL Multiphysics to further understand the effect of microstructures on sensing performance, as shown in Figure 5e. Compared to the planar structure, it is clear to see that there is a greater stress concentration on the tip of the micropattern at an external pressure of 5 kPa (the maximum contact stress of 41.2 kPa and 17180 kPa for the non-micropatterned and the micropatterned, respectively). Meanwhile, the ratio of change in height (Δd/d) for the micropatterned sensor is also more evident than that of the planar sensor (Table S2). Thus, the use of the micropatterned structure also effectively improves the performance of the capacitive sensor.

### 3.5. Sensing Applications of the Bioinspired Sensor

**Figure 6:**
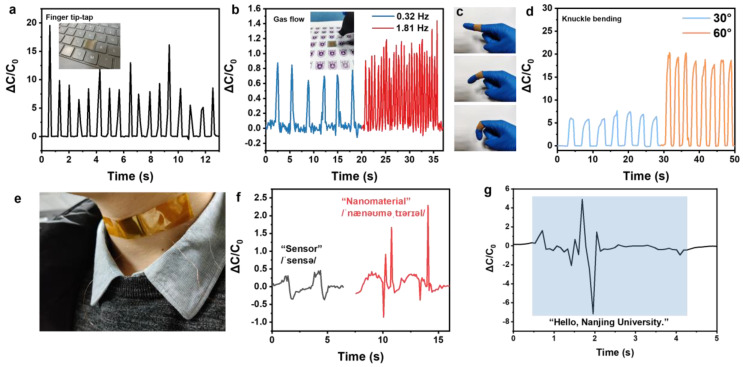
A series of human-machine interaction applications of the capacitive pressure sensor (**a**) Capacitance response when the fingertip-top on the sensor. Inset: Photograph of the sensor attached to the keyboard. (**b**) Capacitance response of non-contact sensing. Inset: photograph of the airflow generated by a rubber suction bulb at a frequency of 0.32 Hz and 1.81 Hz. (**c**) Photograph of the sensor attached to the joint of the index finger with adhesive bandage. (**d**) Dynamic response of joint bending. (**e**) Photograph of the sensor attached to the throat. (**f**,**g**) Real-time response of the sensor when pronouncing the words “sensor” and “nanomaterial” twice, respectively, and as well as a short sentence “Hello, Nanjing University.”

Owing to the basic mechanism and excellent sensing behaviors of the MXene/P(VDF-TrFE-CFE) sensor, a few demonstrations of real-time physiological applications are shown in Figure 6. The prepared sensor can serve as a keyboard and shows a stable and synchronous response as a finger clicks on it (Figure 6a). Typically, the pressure generated by the airflow is particularly low, so airflow of different frequencies is applied to the device by using a rubber suction bulb (Figure 6b), which further confirms that the device not only has a good detection limit but also has an excellent resolution to time. Thus, this non-contact sensing mode will play an important role in weather forecasts. In addition, the flexible sensor can be used for the daily monitoring of the human body. Figure 6c displays the device attached to knuckles with an adhesive bandage. The corresponding response of the device exhibits significantly differently as the flexion angle of the knuckles is different (Figure 6d). When bent at a large angle, the device generates a stronger signal because of the greater force exerted, causing the increase of effective capacitance. Therefore, this approach can be considered an effective way for assessments of muscle or neurological rehabilitation. We further investigate voice sensing via monitoring the vibrations of the vocal cords, as shown in Figure 6e. When we pronounce “sensor” or “nanomaterial” twice, the same word shows a similar response, which is important for signal repeatability. Notably, contrary to a simple word “sensor”, a compound word such as “nanomaterial” exhibits more signal peaks (Figure 6f). It is “nanomaterial” that consists of multiple syllables while “sensor” only has two syllables. In addition to words, sentences can also be distinguished by the device. For example, the sentence “Hello, Nanjing University.” shows a series of continuous characteristic signals (Figure 6g), which exhibits great potential in human-machine interfacing systems.

## 4. Conclusions

In summary, we demonstrate a flexible capacitive pressure sensor inspired by the structure of human skin via simple assembly. Briefly, the high elastic micropatterned PDMS with electrodes is constructed by using easily available and recyclable abrasive paper; and MXene/P(VDF-TrFE-CFE) nanocomposite is chosen as the dielectric layer to enhance the sensitivity of the device. The prepared MXene-based capacitive pressure sensor presents a high sensitivity with great linearity (16.0 kPa^−1^ for less than 10 kPa), a low detection limit of 8.9 Pa, and a relatively fast response time within 229 ms. A high dielectric permittivity layer is created by using MXene nanosheets. The sensor could detect series of dynamic physiological signals such as typing, knuckle motion, and vibrations of the vocal cords. Overall, it will bring great application prospects to the field of wearable technologies and human-machine interfacing systems.

## Data Availability

The data presented in this study are available on request from the corresponding author.

## Supplementary Materials

The following are available online at https://www.mdpi.com/2079-4991/11/3/716/s1, Figure S1: Schematic illustration describing the formation of the pressure sensor, Figure S2: SEM image of Ti3AlC2 (MAX phase), Figure S3: (a) 3D morphology of the micropatterned PDMS in an area of 1 × 1 mm^2^. (b) Height profile of cross section of the micropatterned PDMS, Table S1: 3D roughness parameters of the micropatterned PDMS, Table S2: Calculation results in Finite-element simulation at an external load pressure of 5 kPa.
