# Supplementary material for: Skin-Inspired Pressure Sensor with MXene/P(VDF-TrFE-CFE) as Active Layer for Wearable Electronics
Source: Nanomaterials (Basel). 2021 Mar 12;11(3):716. doi: 10.3390/nano11030716 (PMC7999094; doi:10.3390/nano11030716)
Supplement: Supplementary file 1 [file nanomaterials-11-00716-s001.pdf]

Supporting Information

Skin-Inspired Pressure Sensor with MXene/P(VDF-TrFE-CFE) as Active Layer for Wearable Electronics

Xiao-Quan Shen, Ming-Ding Li, Jun-Peng Ma and Qun-Dong Shen *

Department of Polymer Science & Engineering and Key Laboratory of High Performance Polymer Materials & Technology of MOE, School of Chemistry & Chemical Engineering, Nanjing University, Nanjing 210023, China; xqshennju@163.com (X.-Q.S.); mingdingli2016@gmail.com (M.-D.L.); 739182102@qq.com (J.-P.M.)

* Correspondence to qdshen@nju.edu.cn (Q.-D.S.)

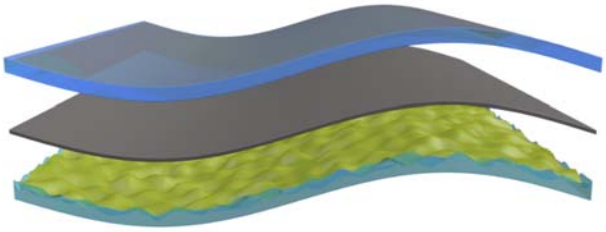

Figure S1. Schematic illustration describing the formation of the pressure sensor.

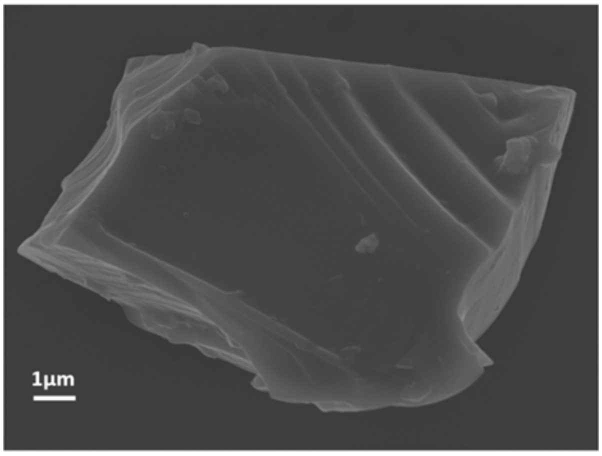

Figure S2. SEM image of Ti_3AlC_2 (MAX phase).

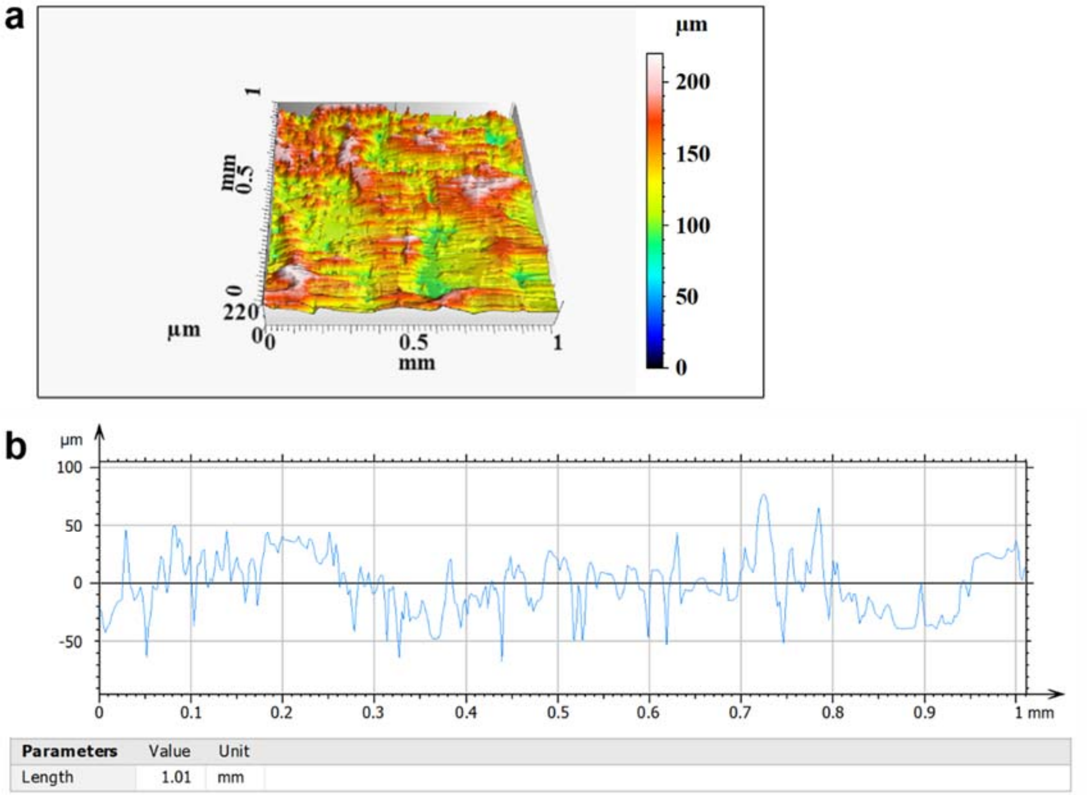

Figure S3. (a) 3D morphology of the micropatterned PDMS in an area of 1×1 mm^2. (b) Height profile of cross section of the micropatterned PDMS.

Table S1. 3D roughness parameters of the micropatterned PDMS.

ISO 25178		
**Height Parameters**		
Sq	28.8	µm
Ssk	0.134	
Sku	2.93	
Sp	80.9	µm
Sv	139	µm
Sz	220	µm
Sa	23.4	µm

ISO 4287			
**Amplitude parameters - Roughness profile**			
Rp	65.2	µm	*Gaussian filter, 0.25 mm*
Rv	53.6	µm	*Gaussian filter, 0.25 mm*
Rz	119	µm	*Gaussian filter, 0.25 mm*
Rc	58.0	µm	*Gaussian filter, 0.25 mm*
Rt	119	µm	*Gaussian filter, 0.25 mm*
Ra	13.3	µm	*Gaussian filter, 0.25 mm*
Rq	17.7	µm	*Gaussian filter, 0.25 mm*
Rsk	0.364		*Gaussian filter, 0.25 mm*
Rku	4.63		*Gaussian filter, 0.25 mm*
**Material Ratio parameters - Roughness profile**			
Rmr	0.294	%	*c = 1 µm under the highest peak, Gaussian filter, 0. ...*
Rdc	26.4	µm	*p = 20%, q = 80%, Gaussian filter, 0.25 mm*

Table S2. Calculation results in Finite-element simulation at an external load pressure of 5 kPa.

	Maximum contact stress/kPa	Ratio of change in height/%
Non-micropatterned	41.2	0.05
Micropatterned	17180.4	28.26
